# Pharmacological effects of berberine on mood disorders

**DOI:** 10.1111/jcmm.13930

**Published:** 2018-11-18

**Authors:** Jie Fan, Kun Zhang, Yang Jin, Bingjini Li, Shuohui Gao, Jiaming Zhu, Ranji Cui

**Affiliations:** ^1^ Jilin Provincial Key Laboratory on Molecular and Chemical Genetic Second Hospital of Jilin University Changchun China; ^2^ Department of Gastrointestinal Colorectal Surgery China‐Japan Union Hospital of Jilin University Changchun China

**Keywords:** anxiety, berberine, bipolar disorder, depression, mechanism, pharmacology

## Abstract

Berberine, a natural isoquinoline alkaloid, is used in herbal medicine and has recently been shown to have efficacy in the treatment of mood disorders. Furthermore, berberine modulates neurotransmitters and their receptor systems within the central nervous system. However, the detailed mechanisms of its action remain unclear. This review summarizes the pharmacological effects of berberine on mood disorders. Therefore, it may be helpful for potential application in the treatment of mood disorders.

## 1 INTRODUCTION

Mood disorders are common, chronic, recurrent mental illnesses that affect millions of individuals worldwide.^1^, ^2^ The primary mood disorders are major depressive disorder and bipolar disorder. Most patients with mood disorders receive some benefit from available treatments.^3^, ^4^ However, full remission of clinical symptoms is rarely achieved owing to complex pathophysiology. Moreover, many classes of antidepressant have serious side‐effects such as drowsiness, dryness of mouth, headache, nausea, and sexual dysfunction.^5^, ^6^ There is, therefore, an urgent need to develop alternative drugs. Berberine is an herbal drug used in traditional Chinese medicine that has recently been shown to alleviate mood disorders in a number of ways.^7^, ^8^ Berberine, therefore, has the potential to become a mainstream drug for treating mood disorders. This article reviews the literature on the pharmacological effects of berberine in the treatment of various mood disorders. We focus on underlying mechanisms and pathways that mediate the multiple pharmacological actions of berberine. The applicability of berberine to mood disorders is also discussed in this review.

## 2 BERBERINE

Berberine, a natural isoquinoline alkaloid, is widely used in traditional Chinese medicine.^9^ It is isolated from several herbal species, including *Berberis Hydrastis canadensis* (goldenseal), *Xanthorhiza simplicissima* (yellow root), *Phellodendron anureses* (Amur cork tree), *Coptis chinensis* (Chinese goldthread), *Tinospora cordifolia*,* Argemone mexicana* (prickly poppy), and *Eschscholzia californica* (Californian poppy).^10^ However, it has poor bioavailability, which seriously limits its application and development.^11^ It is an intense yellow powder, odourless with a characteristic alkaloidal bitter taste. It is very slightly soluble in water and ethanol and is sparingly soluble in methanol. The chloride or sulphate salts of berberine are relatively more soluble and are used clinically.^12^ Berberine has multiple therapeutic actions, including antioxidant, anti‐inflammatory, antitumour, antimicrobial, hepatoprotective, hypolipidemic, and hypoglycemic actions.^13^ The pharmacology of berberine is well documented; however, there is renewed interest in berberine because of its benefits in various neurodegenerative and neuropsychiatric disorders.^7^ Wang et al reported that berberine could easily cross the blood‐brain barrier on systemic administration^14^ and recent studies show that berberine has a protective effect on central nervous system disorders, such as Alzheimer's, cerebral ischaemia, mental depression, schizophrenia, and anxiety.^7^, ^12^, ^15^ Here, we review the pharmacology of berberine in detail and highlight its efficacy in the treatment of mood disorders.

## 3 PHARMACOLOGICAL EFFECT OF BERBERINE ON DEPRESSION

### 3.1 The effect of berberine on neurotransmitters in depression

The original monoamine hypothesis postulates that the dysregulation of serotonin, norepinephrine, and dopamine has been theorized to be a core pathogenic factor in depression. Dysregulation of neurotransmitters can cause depression.^16^ Berberine can regulate brain neurotransmitters, especially biogenic amines.^12^, ^17^ Norepinephrine (NE), serotonin (5‐HT) and dopamine (DA) are neurotransmitters released from neurons during synaptic transmission in the nervous system.^12^ The studies demonstrated that berberine (10, 20 mg/kg, p.o.), dramatically reduced the immobility time during the forced swim test and the tail suspension test.^18^ Either acute or chronic administration of berberine at low doses results in increased levels of NE, 5‐HT, and DA in whole‐brain samples. Kulkarni et al showed that acute administration of berberine (5 mg/kg, i.p.) in mice caused increased levels of norepinephrine (31%), serotonin (47%), and dopamine (31%). Chronic administration of berberine (5 mg/kg, ip) for 15 days significantly increased the levels of norepinephrine (29%), serotonin (19%) as well as dopamine (52%).^12^ These increases in biogenic amine levels are attributed to the inhibition of monoamine oxidase by berberine.^19^, ^20^ Berberine can also inhibit the release of NE via activation of adrenergic α2 autoreceptors^21^ and can affect DA in a manner that antagonizes D2 and agonizes D1 receptors.^22^, ^23^ Substance P shows a strong negative correlation with serum concentrations of the primary 5‐HT metabolite, 5‐hydroxyindoleacetic acid.^24^ Berberine reverses the increase in substance P levels induced by reserpine in the cerebral cortex and hippocampus.^25^

Sigma receptors play an important role in the modulation of various neurotransmitters. Sigma ligands can modulate the activity of the neurotransmitter systems, such as noradrenergic, serotonergic, dopaminergic, and glutamatergic ones.^26^ Meurs et al reported that sigma 1 receptor‐mediated increase in hippocampal extracellular dopamine.^27^ Moreover, some sigma agonists are found to have antidepressant‐like activity perhaps with fewer side‐effects.^28^ These receptors are a promising therapeutic target for neuropsychiatric disorders, in particular, for depression. Recent studies have provided further evidence for the involvement of sigma receptors in the pathophysiology of major depression psychiatric disturbances.^28^, ^29^, ^30^, ^31^ Berberine affects sigma receptor 1 similarly to many antidepressant drugs^32^ indicating its potential for the treatment of major depression.

### 3.2 The effect of berberine on antioxidation in depression

Clinical studies of patients with depression have shown disturbances to oxidation, such as elevated lipid peroxidation products and reduced levels of superoxide dismutase.^33^ Patients with depression also accompanied changes in brain volume. Increases in reactive oxygen species (ROS) and decreased antioxidant defences may cause oxidative modifications of proteins and DNA. The damage of stability of proteins and DNA may result in apoptois, and in part explain the brain volumetric changes evident in depression.^34^ Arora et al found that treatment with berberine (10 and 20 mg/kg) produced a significant reduction in lipid peroxide levels in the cerebral cortex of reserpine administered rats.^25^ Berberine inhibits the generation of ROS by suppressing overexpression of the nicotinamide adenine dinucleotide phosphate oxidase (NOX) enzyme complex.^35^, ^36^ Meanwhile, berberine treatment restored the levels of nonprotein thiols, superoxide dismutase and catalase, which were significantly decreased by reserpine in the cerebral cortex and hippocampus.^25^, ^37^

Lukic et al provided evidence that depression is characterized by up‐regulation of nuclear transcription factor‐κB (NF‐κB). Major depressive disorder subjects exhibited higher levels of NF‐κB compared to controls.^38^ NF‐κB activity is regulated at least in part by the intensity of intracellular oxidative stress.^39^, ^40^ Li et al reported that NF‐κB can be activated by oxidative stress (such as by exposure to H_2_O_2_).^41^ Berberine interacts directly with nucleic acids, and with several proteins, including p53, NF‐κB, and oestrogen receptors.^42^, ^43^, ^44^ Arora et al also observed a significant increase in levels of NF‐κB and caspase‐3 in the cerebral cortex and hippocampus of reserpine‐treated rats and treatment with berberine down‐regulated the elevated levels of NF‐κB and caspase‐3.^25^ Chronic berberine treatment inhibited NF‐κB signalling pathway in the hippocampus and prevented the depressive deficits both in sucrose preference test and novelty‐suppressed feeding test in mice induced by chronic unpredictable mild stress.^45^ These studies indicate that berberine may be of use as an antidepressant through the NF‐κB signalling pathway, which may be activated by oxidative stress.

### 3.3 The effect of berberine on nitric oxide synthesis in depression

Systemic inhibition of nitric oxide synthase induces antidepressant‐like effects in the rat hippocampus. The neuronal nitric oxide synthase inhibitor significantly decreased immobility time.^46^ Pharmacological manipulation of nitric oxide pathway by berberine can be observed in a reserpine‐induced model of depression. Berberine (5 and 10 mg/kg, ip) reversed the increased of immobility period induced by reserpine.^12^ Evidence has suggested that neuronal nitric oxide synthase inhibition increases serotonin signalling and activaties prosencephalic 5HT1A receptors.^47^ There is also a close connection between adenosine monophosphate‐activated protein kinase (AMPK) pathway and nitric‐oxide synthesis. AMPK plays an important role in regulating NO synthesis in endothelial cells.^48^ The activity of AMPK pathway is regulated by berberine.^49^ AMPK is an upstream kinase of endothelial nitric oxide synthase (eNOS) that promotes eNOS phosphorylation, complex formation between eNOS and heat shock 90 kDa protein, and nitric oxide (NO) production.^50^, ^51^ Recent evidence has shown that reduced nitric oxide levels can induce antidepressant‐like effects.^50^ Moreover, the L‐arginine‐NO‐cyclic guanosine monophosphate signalling pathway is important in the antidepressant action of berberine chloride.^46^ Excessive levels of cyclic guanosine monophosphate can produce a depression‐like state, while reduced levels can produce antidepressant‐like actions.^52^

### 3.4 The effect of berberine on neuroinflammation in depression

Neuroinflammation may have a role in the pathogenesis of depression.^37^, ^53^ Inflammation‐associated disorder of serotonergic and glutamatergic neurotransmission ultimately induces depression‐like behaviour.^54^ Mice induced by chronic unpredictable mild stress display enhanced levels of pro‐inflammatory cytokines, including interleukin‐6, interleukin‐1‐beta, and tumour necrosis factor β in hippocampus. Then the increased pro‐inflammatory cytokines were decreased by orally administration berberine.^45^ In addition, treatment with berberine attenuated the increased levels of the interleukin‐1‐beta in reserpine‐treated rats.^25^ It has been suggested that pro‐inflammatory cytokines may affect the catabolism and disposition of various neurotransmitters through activation of indoleamine 2,3‐dioxygenase (IDO).^55^, ^56^ Increased IDO activity may also decrease tryptophan availability, impacting serotonergic neurotransmission.^57^ Berberine, a newly identified IDO inhibitor, significantly decreased the production of kynurenine in A549 cells.^58^ Increased kynurenine may metabolize to neurotoxic metabolites, such as quinolinic acid, thereby, influencing glutamatergic neurotransmission.^57^

### 3.5 The effect of berberine on neurotrophic factors in depression

Nerve growth factor plays a role in the modulation of synaptic function and plasticity in the CNS.^58^ Berberine potentiates nerve growth factor (NGF) activity, which can increase NGF‐induced neurite outgrowth in a dose‐dependent manner.^59^ In some depression models, berberine decreased ROS levels,^36^ and increased NGF‐mediated neurite outgrowth via the phosphoinositide 3‐kinase/protein kinase B/nuclear factor‐E2‐related factor 2‐dependent pathway.^36^ In addition, berberine has a neuroprotective effect in a dose‐dependent manner, low‐dose berberine significantly increased cell viability, while high‐dose berberine inhibited cell viability.^60^

An antidepressant effect of berberine results from elevation of brain‐derived neurotrophic factor (BDNF) levels.^17^, ^61^ Bombi et al reported that berberine restored the decreased level of BDNF mRNA in the rat hippocampus following withdrawal from repeated morphine injection.^17^ Our recent study indicated that berberine exerts antidepressant‐like effects in ovariectomized mice, partly through the effects of berberine on BDNF‐ cyclic adenosine monophosphate (cAMP) response element‐binding protein and eukaryotic elongation factor 2 (eEF2) pathways.^61^ The BDNF‐ cAMP‐response element binding protein (CREB) pathway is a well‐established antidepressant pathway which is critical for antidepressant action. Furthermore, eEF2 is involved in the actions of rapid‐onset antidepressants.^62^, ^63^ The reductions of hippocampal BDNF and phosphorylated eEF2 levels in ovariectomized mice are reversed by berberine treatment.^61^ These studies suggest that berberine may rapidly produce antidepressant‐like behaviour.

### 3.6 The effect of berberine on hormonal regulation in depression

Hormonal imbalance can cause a variety of neurological disorders. One of the most common examples is the link between diabetes and major depressive disorder.^64^, ^65^ A growing number of studies have demonstrated that berberine can affect mood by regulating plasma corticosterone levels. Palmatine, a quaternary protoberberine alkaloid, produced antidepressant‐like activity by decreasing plasma corticosterone levels.^66^ *Phellodendron*, which is rich in berberine, reduced the effects of cortisol exposure and perceived daily stress.^67^ Treatment with berberine attenuated the depressive‐like behaviour induced by repeated corticosterone injection.^68^ Fluctuations in gonadal hormone levels are believed to contribute to these depressive conditions.^69^ Although, some studies show an effect of berberine on gonadal hormone levels, it is not known whether the antidepressant effects of berberine involve gonadal hormonal regulation.

### 3.7 New perspectives of berberine action in depression

Studies in humans have shown an association between irritable bowel syndrome and depression.^70^ Zhu et al investigated the mechanism of berberine by examining alterations to gastrointestinal tract histopathology and the gut flora profile in a chronic mild stress rat model. Berberine reversed the physical damage brought about by stress within the gastric mucosa and intestinal microvilli of the stomach, ileum, caecum, and colon.^71^ This study showed that high concentrations of berberine can protect rats from various symptoms of chronic stress and depression, indicating its potential clinical use.

### 3.8 The effect of berberine on bipolar affective disorder

In recent years, prolyl oligopeptides (POPs) have gained prominence as targets for the treatment of bipolar affective disorder.^72^ POP has been reported to participate in the processing of neuropeptide precursors.^73^ Moreover, neuroprotective effects of POPs inhibitors have been reported in experimental animals.^74^, ^75^ Berberine inhibits POPs in a dose‐dependent manner.^72^ However, few studies have reported the effects of berberine in bipolar disorder. As noted below, some neurotransmitters such as dopamine, glutamate, and γ‐aminobutyric acid (GABA) are responsible for mood cycling, while, dopamine and glutamate increase transmission during the manic phase.^76^, ^77^ More evidence is required to substantiate a relationship between bipolar affective disorder and berberine.

## 4 PHARMACOLOGICAL EFFECT OF BERBERINE ON ANXIETY

Anxiety is an aversive emotional state that affects approximately one‐eighth of the worldwide population.^78^ A significant anxiolytic effect of berberine can be observed in the elevated plus‐maze test. Berberine increased the time spent in and the exploration of the open arms, and decreased the entries to and time spent in the closed arms.^17^, ^79^

It is worth noting that berberine regulates biogenic amines in a concentration‐dependent manner. At a low dose, berberine (10 and 20 mg/kg) is effective in depression by increasing levels of NE, 5‐HT and DA.^18^ In contrast, high doses of berberine (100, 500 mg/kg) decreased concentrations of biogenic amines.^18^, ^79^ Furthermore, berberine increased the concentrations of their metabolites in the brain stem. The anxiolytic mechanism of berberine might be related to the increased turnover rates of monoamines in the brain stem and decreased serotonergic system activity. Moreover, berberine decreased serotonergic system activity via activation of somatodendritic 5‐HT1A autoreceptors and inhibition of post‐synaptic 5‐HT1A and 5‐HT2 receptors.^79^ Berberine also has an inhibitory effect on glutamate receptors and can reduce glutamate, 5‐HT and NE levels.^80^, ^81^

Extensive comorbidity among depressive disorders and anxiety disorders indicates related disease etiologies.^82^, ^83^ Dysregulation of GABA in anxiety has been reported in a number of studies.^84^ Benzodiazepines, GABAA receptor agonists, are commonly used in the clinical treatment of anxiety. Berberine alkaloids bind with the high‐affinity benzodiazepine site on the GABAA receptor.^84^, ^85^ Anxiety research has predominantly focused on the neurotransmitter systems, including GABAergic and serotoninergic systems. However, Kuloglu et al recently established a link between oxidative stress and certain anxiety disorders, demonstrating that other systems, such as oxidative metabolism, can affect the regulation of anxiety.^86^ Although the antioxidant effect of berberine has been confirmed,^87^ it has not been reported in animal models of anxiety. Further studies are warranted to validate this link and to understand the pathogenic mechanisms involved.

## 5 TOLERABILITY AND SAFETY

Berberine has been used in the clinic for several decades.^88^, ^89^ It displays various pharmacological effects, including efficacy against gastroenteritis, abdominal pain, and diarrhoea. It also has anti‐microbial, anti‐diabetic, and anti‐inflammatory properties.^88^, ^90^ Chronic administration of berberine (1200‐2000 mg/d) for at least 2 months significantly decreased total cholesterol levels and low‐density lipoprotein cholesterol without major adverse effects.^91^ Yin et al and Dong et al studied the anti‐diabetic properties of berberine (500‐1500 mg daily for 3 months) 92 and they hypothesized that berberine induces a significant reduction in postprandial glucose levels. In addition, in patients randomized to receive 800 mg of berberine hydrochloride daily for 2 months, a trend of improvement was observed for IBS symptom scores compared with placebo.^74^

Berberine does not display any genotoxic, cytotoxic, or mutagenic activity.^7^ However, berberine has remarkable cytotoxicity on a wide range of cancer cell lines because of its protoberberine skeleton.^73^ Standard doses of berberine are usually well‐tolerated and adverse reactions are rare. In contrast, high doses have been associated with arterial hypotension, dyspnoea, flu‐like symptoms, gastrointestinal discomfort, constipation, and cardiac damage.^93^ Yin et al reported that approximately 34.5% of patients treated with berberine (500 mg three times daily) experienced transient adverse gastrointestinal effects.^92^ Moreover, berberine can lower blood sugar levels and blood pressure and should, therefore, be used with caution in people with diabetes or low blood pressure.^94^, ^95^ Berberine is a potent displacer of bilirubin when tested in vitro using plasma from jaundiced new‐born babies and, therefore, poses a risk of kernicterus.^96^ Its use should, therefore, be avoided in jaundiced infants and pregnant woman, even in small doses. Although berberine has not been administered to patients with depression, as a traditional Chinese herbal medicine, berberine has been used in the East for hundreds of years. However, berberine targets are involved in a wide range of molecular activities and can alter many pathological states. Further research is required to test whether berberine is a promising candidate for the treatment of mood disorders.

## 6 CONCLUSION

**Figure 1:**
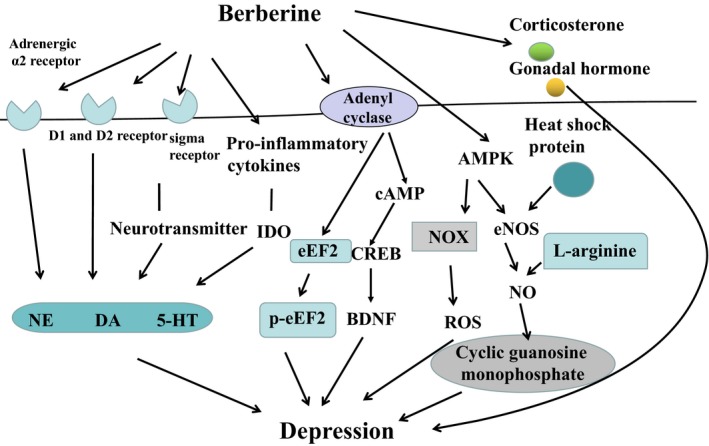
Schematic representation of molecular mechanism of berberine involved with depression. The schematic drawing showed possible regulatory network of berberine in depression. Black arrows indicate stimulation. Some mechanisms are indicated by abbreviations. D1: dopamine 1 receptor; D2: dopamine 2 receptor; NE: norepinephrine; 5‐HT: serotonin; DA: dopamine; ROS: reactive oxygen species; NOX: nicotinamide adenine dinucleotide phosphate oxidase; AMPK: adenosine monophosphate‐activated protein kinase; eNOS: endothelial nitric oxide synthase; NO: nitric oxide; IDO: indoleamine‐ 2,3‐dioxygenase; BDNF: brain‐derived neurotrophic factor; eEF2: eukaryotic elongation factor 2; cAMP: cyclic adenosine monophosphate; CREB: cAMP‐response element binding protein

Based on the published findings, we conclude that berberine may be a potential treatment for mood disorders. Berberine in various mood disorders and its mechanism of action are summarized in Table 1. Schematic representation of the mechanism of berberine involved with depression is shown in Figure 1. Further research into the safety profile of berberine is required before its clinical application.

**Table 1:** Berberine in various mood disorders and its mechanism of action

Mood disorders	Mechanism	References
Depression	Inhibition of monoamine oxidase activity	19, 20
	Increase of NE, 5‐HT and DA levels	12
	Inhibition of α2 auto receptors	21
	Antagonism of D2 receptors and agonism of D1 receptors	22, 23
	Involvement with substance P	25
	Involvement with sigma receptor	32
	Inhibition of NOX and ROS	35, 36
	Reduction of lipid peroxide and superoxide dismutase levels	33
	Involvement of the l‐arginine‐NO‐cGMP pathway	46
	Involvement with tumour necrosis factor β, interleukin‐6, interleukin‐1‐beta, IDO, kynurenine levels	45, 55, 56
	Induction of NGF secretion	59
	Activation of phosphoinositide 3‐kinase/protein kinase/nuclear factor‐E2‐related factor 2‐mediated regulation	36
	Activation of BDNF‐cAMP response element‐binding protein and eEF2 pathways	61
	Protect gastrointestinal tract	71
	Decrease of plasma corticosterone levels	66
	Fluctuations in gonadal hormone levels	69
Bipolar disorder	Activation of POP	72
Anxiety	Decrease of 5‐HT, NE, and DA levels	18
	Binding with GABAA receptor	84, 85
	Inhibition of glutamate receptors	80, 81
	Increase in turnover rates of monoamines	79
	Activation of 5‐HT1A and inhibition of 5‐HT1A and 5‐HT2 receptors	79

## CONFLICTS OF INTEREST

The authors declare no conflict of interest.
